# Glucose-regulated protein 78 is essential for cardiac myocyte survival

**DOI:** 10.1038/s41418-018-0109-4

**Published:** 2018-04-17

**Authors:** Xiaoding Wang, Xukun Bi, Guangyu Zhang, Yingfeng Deng, Xiang Luo, Lin Xu, Philipp E. Scherer, Anwarul Ferdous, Guosheng Fu, Thomas G. Gillette, Amy S. Lee, Xuejun Jiang, Zhao V. Wang

**Affiliations:** 1 Department of Cardiology, Renmin Hospital of Wuhan University, Wuhan, Hubei China; 2 Division of Cardiology, Department of Internal Medicine, University of Texas Southwestern Medical Center, Dallas, TX USA; 3 Department of Cardiology, Biomedical Research (Therapy) Center, Sir Run Run Shaw Hospital, School of Medicine, Zhejiang University, Hangzhou, Zhejiang China; 4 Department of Cardiology, Zhongnan Hospital of Wuhan University, Wuhan, Hubei China; 5 Touchstone Diabetes Center, Department of Internal Medicine, University of Texas Southwestern Medical Center, Dallas, TX USA; 6 Department of Biochemistry and Molecular Medicine, USC Norris Comprehensive Cancer Center, Keck School of Medicine, University of Southern California, Los Angeles, CA USA

## Abstract

Secretory and transmembrane proteins rely on proper function of the secretory pathway for folding, posttranslational modification, assembly, and secretion. Accumulation of misfolded proteins in the endoplasmic reticulum (ER) stimulates the unfolded protein response (UPR), which communicates between the ER and other organelles to enhance ER-folding capacity and restore cellular homeostasis. Glucose-regulated protein of 78 kDa (GRP78), an ER-resident protein chaperone, is a master regulator of all UPR signaling branches. Accumulating studies have established a fundamental role of GRP78 in protein folding, ER stress response, and cell survival. However, role of GRP78 in the heart remains incompletely characterized. Here we showed that embryos lacking GRP78 specifically in cardiac myocytes manifest cardiovascular malformations and die in utero at late gestation. We went further to show that inducible knockout of GRP78 in adult cardiac myocytes causes early mortality due to cardiac cell death and severe decline in heart performance. At the cellular level, we found that loss of GRP78 increases apoptotic cell death, which is accompanied by reduction in AKT signaling and augmentation of production for reactive oxygen species. Importantly, enhancing AKT phosphorylation and activity leads to decreases in oxidative stress and increases in cardiac myocyte survival. Collectively, our results demonstrate an essential role of GRP78 in ensuring normal cardiogenesis and maintaining cardiac contractility and function.

## Introduction

Approximately one third of human proteins are either secretory or transmembrane [1]. Proper folding and multiunit complex formation are essential for these molecules to communicate between tissues, relay signaling cascades, and ensure functionality of the cell. Physiological fluctuations and pathological alterations adversely affect secretory or transmembrane protein function via triggering protein misfolding in the endoplasmic reticulum (ER). The cell has evolved a deliberate system to counteract protein-folding stress. This so-called ER stress response is part of the unfolded protein response (UPR), which is an evolutionarily conserved pathway to accommodate protein-folding stress and restore cellular homeostasis [2].

ER stress response is governed by three distinct signaling branches [3]. The master ER protein chaperone glucose-regulated protein 78 kDa (GRP78) binds the ER luminal domains of PERK (protein kinase RNA-like ER kinase), IRE1 (inositol-requiring protein 1), and ATF6 (activating transcriptional factor 6) [4, 5]. This interaction retains the UPR at resting conditions. Upon accumulation of misfolded proteins in the ER, GRP78 preferably associates with the hydrophobic patches of malfolded polypeptides and therefore releases the sequestration of these three transducers [6]. Different mechanisms then ensue to stimulate the ER stress response. Dimerization of PERK triggers autophosphorylation and phosphorylates eIF2α, which attenuates translation initiation, reduces ER luminal load, and creates a temporal window for repair. IRE1, upon activation, manifests an endoribonuclease activity to target multiple substrates [7]. The most studied one, X-box binding protein 1 (XBP1), can be cleaved at mRNA level. The resulting spliced XBP1 (XBP1s) encodes a potent transcriptional factor involved in ER chaperone stimulation and ER-associated protein degradation [8, 9]. In contrast, ATF6 is translocated to the Golgi after liberation from the ER, where ATF6 undergoes regulated intramembrane proteolysis [10]. The cytosolic domain ATF6 then migrates to the nucleus and functions as a transcriptional factor [11, 12]. These three signaling branches coordinate with each other and orchestrate to restore cellular homeostasis [13–17]. Under persistent stress, however, apoptosis may ensue to eliminate permanently injured cells.

Emerging evidence suggests that GRP78 plays essential roles in development and cell survival [5]. Deletion of GRP78 causes defects in gestation and embryonic lethality at 3.5 days post coitum [18, 19]. Tissue-specific knockout of GRP78 leads to cell death in respective cell types, including adipocytes [20] and lung epithelial cells [21]. Recent studies indicate that GRP78 governs cardiac myocyte phenotype and function under various pathological conditions [22]. Myocardial infarction stimulates GRP78 expression in the heart [23]. Importantly, induction of GRP78 via forced overexpression of nuclear ATF6 confers strong cardioprotection against ischemic insult [24]. Even brief upregulation of GRP78 by pharmacological UPR inducers protects the heart from subsequent lethal ischemia [25]. Additionally, we have shown that cardiac ischemia/reperfusion leads to strong upregulation of the UPR and stimulates GRP78 expression at both in vivo and in vitro levels [8]. Despite these, the role of GRP78 in cardiac physiology remains incompletely understood. Here we sought to investigate the function of GRP78 in the heart using genetically engineered animal models and primary cardiac myocyte culture.

## Results

### Protein secretion from cardiac myocytes

Cardiomyocytes are typically not considered as a traditional secretory cell type. Recent evidence, however, shows that a number of proteins can be released from cardiac myocytes under physiological or pathological conditions [26–28]. Moreover, dysregulation in the secretory pathway in cardiac myocytes plays a causal role in the pathogenesis of various heart diseases [29, 30].

**Fig. 1:**
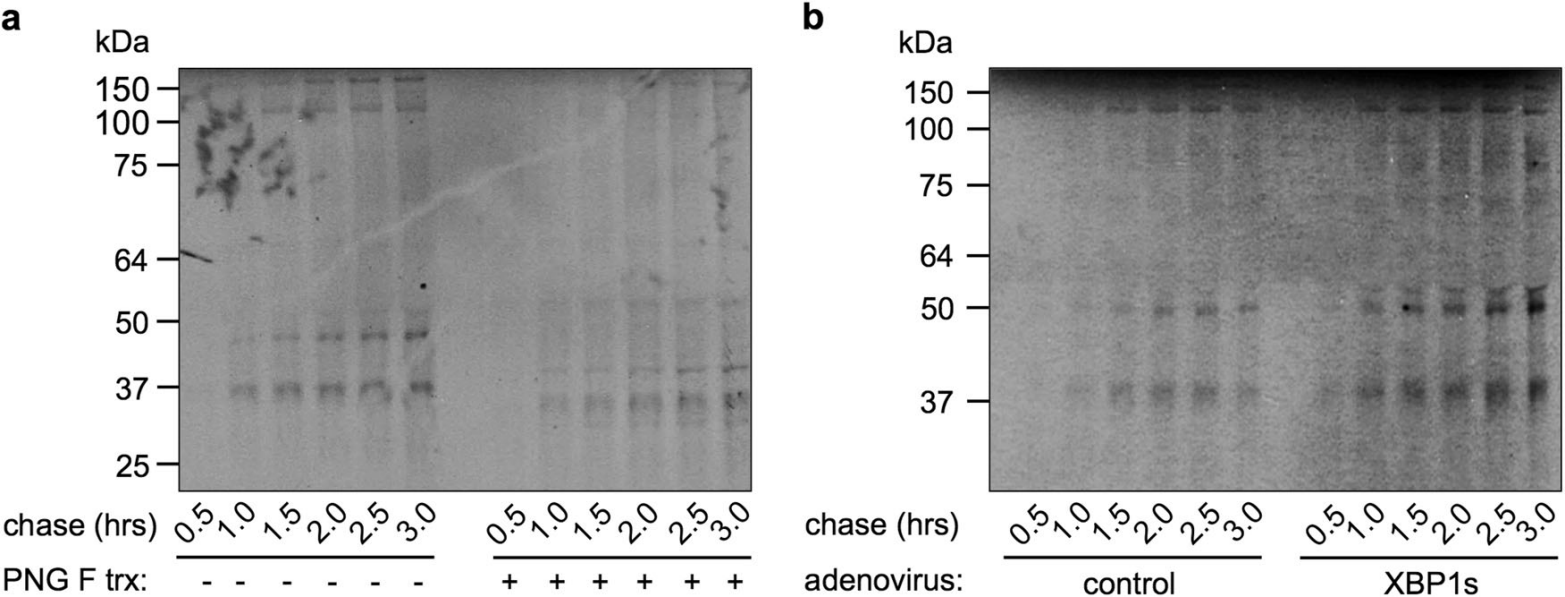
Protein secretion from cardiac myocytes. **a** NRVMs were pulse labeled by ^35^S radioactive amino acids for 15 min. Secretory proteins were chased from culture medium at different times as indicated. A separate aliquot was subjected to PNGase F treatment to remove *N*-linked oligosaccharides. After separation in a SDS-PAGE gel, secreted proteins from cardiac myocytes were visualized by autoradiography. Increases in migration after PNGase F treatment indicate cleavage of glycosylation modifications from secreted proteins. **b** Overexpression of XBP1s, the most conserved branch of the unfolded protein response, led to increases in secreted proteins from cardiomyocytes. NRVMs were first infected by adenovirus expressing either GFP or XBP1s, followed by pulse-chase of secretory molecules

To investigate the functional significance of the secretory pathway in the heart, we first evaluate global protein secretion from cardiac myocytes. Isolated neonatal rat ventricular myocytes (NRVMs) were pulse labeled by ^35^S-radioactive methionine/cysteine for 15 min. Culture medium, which contained secretory molecules from NRVMs, was removed very 30 min for a total of 3 h. These proteins were then separated in sodium dodecyl sulfate-polyacrylamide gel electrophoresis (SDS-PAGE) gels and visualized by autoradiography. To confirm the secretory nature, PNGase F treatment was conducted to remove all *N*-linked oligosaccharides, which are typical modifications of secretory proteins. We found that NRVMs manifested strong secretory capacity (Fig. 1a). Predominant bands showed at 40, 50, and >100 kDa, of which the migration was correspondingly accelerated after PNGase F treatment. This secretory capability of cardiac myocytes could be enhanced by overexpression of a key UPR transcriptional factor XBP1s (Fig. 1b and Supplementary Fig. S1a-c).

### GRP78 expression in cardiac myocytes

**Fig. 2:**
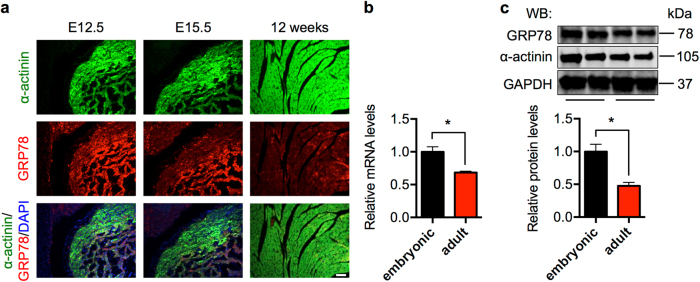
GRP78 expression in cardiac myocytes. **a** Immunofluorescence staining showed GRP78 expression in cardiac myocytes in both embryonic and adult (12 week old) hearts. α-Actinin is a marker for cardiac myocyte. Note that GRP78 is also expressed in α-actinin-negative, non-cardiac tissues. Scale bar: 40 μm. **b** GRP78 expression was decreased in adult compared to embryonic hearts as assessed by qPCR, which was normalized to 18s rRNA. Embryonic indicates 12–15 days post coitum, and adult indicates 3 months old. *N* = 6–7. **c** GRP78 protein level was reduced in the adult heart compared to embryonic stage as shown by immunoblotting. *N* = 6. *p < 0.05

Here we undertook both in vivo and in vitro approaches to determine the role of GRP78 in cardiac myocytes. We first examined the expression profile of GRP78 in the heart. Early studies have shown that GRP78 promoter is active in neonatal hearts and global deletion of GRP78 leads to embryonic lethality [18]. We found that GRP78 was expressed in cardiac myocytes at embryonic days 12.5 and 15.5 (E12.5, E15.5) (Fig. 2a). GRP78 expression was not restricted in the heart, and other tissues had positive signal as well. The expression showed a trend of decrease in adult hearts, which was confirmed at mRNA and protein levels (Fig. 2b, c). Taken together, these results suggest that GRP78 is expressed in cardiac myocytes at high levels in vivo.

### Cardiac-specific deficiency of GRP78 leads to embryonic lethality

To assess the cardiac specific role of GRP78 in vivo, we deleted GRP78 from the heart by crossing the GRP78^fl/fl^ mouse model [18, 19] with the αMHC-Cre transgenic mouse. Since the αMHC promoter is active in cardiomyocytes as early as E8.5 and remains high throughout adulthood, this compound animal model may be used to delineate the role of GRP78 in cardiac development. From the GRP78^fl/fl^ and GRP78^fl/+^;αMHC-Cre breeding, out of 98 pups, we did not identify any live offspring with the genotype of GRP78^fl/fl^;αMHC-Cre while other genotypes were consistent with the Mendelian ratio, suggesting that mice with cardiac myocyte-restricted deletion of GRP78 die in utero.

**Fig. 3:**
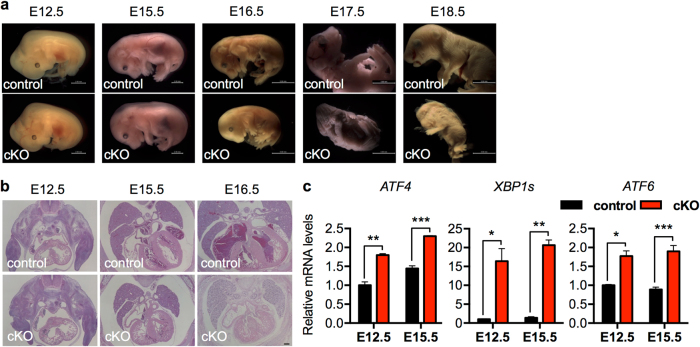
Cardiac-specific deficiency of GRP78 leads to embryonic lethality. **a** GRP78^fl/fl^ mice were bred to the αMHC-Cre transgenic mice to create GRP78 deletion in cardiac myocytes from embryonic day 8.5. Morphological analysis was shown for control and GRP78 cardiac-specific knockout (cKO) embryos isolated at indicated developmental stages. Note that the cKO embryo manifested edema on the back at E15.5, indicating reduced cardiac performance, and died at E16.5. There were no detectable developmental defects in either GRP78^fl/fl^ or GRP78^fl/+^;αMHC-Cre embryos, which were used as controls. Scale bar, 2 mm for E12.5 and E15.5, and 5 mm for E16.5, E17.5 and E18.5. **b** Hematoxylin and eosin (H&E) staining of embryos revealed normal development at E12.5. The cKO heart showed defects in trabeculation at E15.5. H&E signal intensity was decreased in E16.5 cKO, indicating increased proteolysis or decreased protein synthesis. Scale bar: 200 μm. **c** GRP78 knockout caused strong upregulation of markers of the unfolded protein response (UPR). Gene expression of the three signaling branches of the UPR was determined by qPCR from isolated embryonic hearts. *N* = 3–9. *p < 0.05; **p < 0.01; ***p < 0.001

We went further to identify the critical developmental stage affected by loss of GRP78. We isolated embryos from the cross between GRP78^fl/fl^ and GRP78^fl/+^;αMHC-Cre. There were no detectable morphological differences between control (GRP78^fl/+^;αMHC-Cre or GRP78^fl/fl^) and conditional knockout (cKO) (GRP78^fl/fl^;αMHC-Cre) before or at E12.5 (Fig. 3a and data not shown). Compared with control littermates, development of cKO embryos was normal at E15.5, except that they manifested signs of edema. Importantly, cKO embryos of E16.5 showed severe abnormalities with shrunken fetus. Post E17.5 and forward, cKO embryos were not viable and partially dissolved in uterus. Histological analysis revealed defects in trabeculation at the ventricle of E15.5 cKO embryo (Fig. 3b). Consistent with the master role of GRP78 in controlling the UPR [31], three signaling branches were all strongly induced in the GRP78 cKO hearts (Fig. 3c). Collectively, these results suggest that GRP78 plays an essential role in cardiac morphogenesis at the late gestational stage.

### Cardiomyocyte-restricted deletion of GRP78 in adult mice causes early mortality

**Fig. 4:**
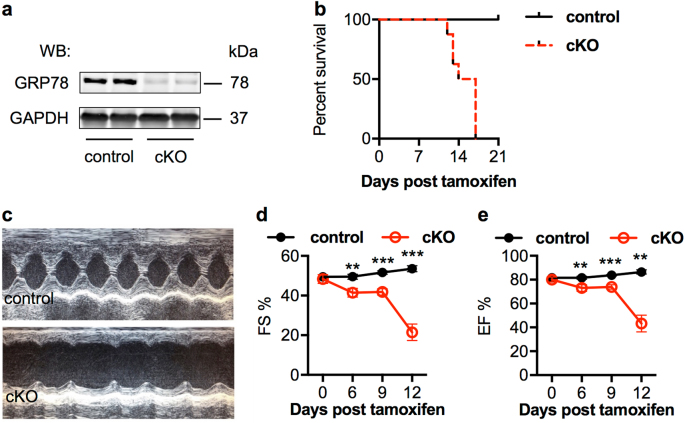
Cardiomyocyte-restricted deletion of GRP78 in adult mice causes early mortality. **a** GRP78^fl/fl^ mice were crossed to the αMHC-MCM mouse model to generate inducible deletion of GRP78 in adult cardiac myocytes. Nuclear translocation of MCM was achieved by tamoxifen injection in adult mice, which effectively eliminated GRP78 in the heart. GAPDH was used as a loading control. **b** GRP78 cKO mice showed 100% mortality within 3 weeks post tamoxifen injection. A significant difference was discovered between control and cKO mice (p < 0.001). The control group included mice of either GRP78 ^fl/fl^ or αMHC-MCM, of which no death was observed. *N* = 7–8. **c** Representative M mode images from echocardiography. **d** GRP78 cKO mice showed progressive decline in cardiac function as shown by significant decreases in FS% (fractional shortening). *N* = 4–11. **e** The EF% (ejection fraction) was reduced in cKO mice compared to controls. *N* = 4–11. **p < 0.01; ***p < 0.001

We next set out to assess the GRP78 role in adult heart by crossing GRP78^fl/fl^ with αMHC-MCM animals. In the double transgenic mice, Cre recombinase is sequestered in cytosol. Upon administration of tamoxifen, Cre is translocated to nucleus to cleave the floxed alleles of GRP78 (Supplementary Fig. S2a). We injected tamoxifen (20 mg/kg body weight, intraperitoneal) for 5 consecutive days. We found GRP78 protein level was strongly decreased only in the cKO hearts (Fig. 4a and Supplementary Fig. S2b).

Importantly, cKO started to show mortality 12 days post the first injection, and eventually all positive animals died within 17 days (Fig. 4b). On the other hand, all control mice, including GRP78^fl/fl^ mice and αMHC-MCM animals, remained alive, indicating that GRP78 is essential for adult cardiac myocyte survival. To further test this, we analyzed cardiac function by echocardiography (Fig. 4c). GRP78 cKO mice showed severe decline of both fractional shortening (Fig. 4d) and ejection fraction (Fig. 4e). A significant reduction was discovered as early as 6 days post tamoxifen treatment, indicating that deficiency of GRP78 in adult cardiac myocytes severely impaired myocyte function and homeostasis.

### Cardiac-specific deficiency of GRP78 leads to more profound pathological cardiac remodeling

We went on to further dissect the mechanism of death of cKO mice. GRP78 deletion in adult cardiac myocytes did not affect body weight (Supplementary Fig. S2c). When sacrificed at day 12 post tamoxifen exposure, cKO mice did not show significant changes in heart weight/body weight ratios (Supplementary Fig. S2d), although the ratios of heart weight/tibia length were slightly decreased (Supplementary Fig. S2e).

**Fig. 5:**
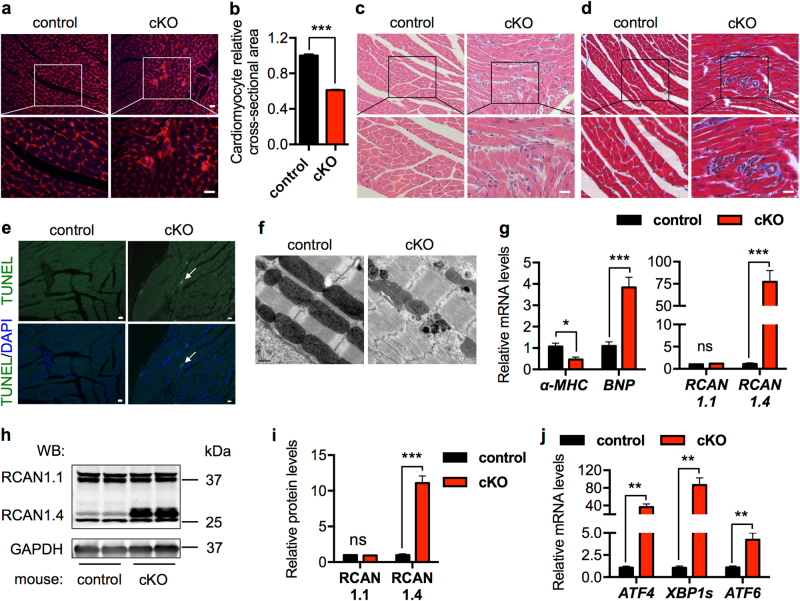
Cardiac-specific deficiency of GRP78 leads to more profound pathological cardiac remodeling. **a** Wheat germ agglutinin (WGA) staining supported that cardiac myocyte size was reduced in the cKO mice, which also showed clusters of non-cardiomyocyte infiltration. Blue indicates nuclei. Scale bar: 20 μm. **b** Quantification of WGA staining from **a** indicated a significant decrease in cardiomyocyte size in the GRP78 knockout myocardium. *N* = 100. **c** GRP78-deficient hearts displayed immune cell infiltration, which were clustered as patches and distributed in the myocardium. Scale bar: 20 μm. **d** Fibrosis was increased in the cKO heart compared to controls. Scale bar: 20 μm. **e** TUNEL staining revealed apoptotic cardiac cell death in the GRP78 cKO heart. Arrow indicates a TUNEL-positive cardiac myocyte. Scale bar: 20 μm. **f** Electron microscopic analysis of the hearts at day 14 post tamoxifen exposure. The cKO mice showed severe sarcomere disarray and mitochondrial disruption at the ultrastructure level. Scale bar: 0.5 μm. **g** GRP78 cKO caused pathological cardiac remodeling as shown by decreases in *α-MHC* and increases in *BNP* expression. Moreover, *RCAN1.4*, a marker of pathological remodeling, was significantly augmented, while the control *RCAN1.1* showed no change. *N* = 6–8. **h** Protein level of RCAN1.4 was greatly stimulated in the GRP78 cKO mice. RCAN1.1 as a control did not differ. GAPDH was used as a loading control. **i** Quantification of **h** showed significant upregulation of RCAN1.4 in the absence of GRP78 in cardiac myocytes. *N* = 6. **j** GRP78 cKO in the heart led to strong induction of the UPR markers. *N* = 6–8. *p < 0.05; **p < 0.01; ***p < 0.001; ns, not significant

At the cellular level, cardiac myocyte size in the cKO mice was significantly smaller than control (Fig. 5a, b). Furthermore, the cKO myocardium showed signs of cardiomyocyte death, distributed over the entire ventricle and typically surrounded by cells with small nuclei (Fig. 5c). These patches of inflammation indicate cardiac cell death with infiltration of immune cells. As a consequence, fibrosis was augmented in the cKO hearts (Fig. 5d). Further, TUNEL (terminal deoxynucleotidyl transferase dUTP nick-end labeling) staining identified apoptotic cardiac cells (Fig. 5e and Supplementary Fig. S2f). Electron microscopy showed that sarcomeres were distorted and mitochondrial morphology was disrupted in the cKO hearts at day 7 post tamoxifen injection (Supplementary Fig. S2g). Sarcomere disarray and mitochondrial abnormalities were further exacerbated a week later (Fig. 5f and Supplementary Figure S2g).

At the molecular level, markers of the fetal gene program were significantly altered (Fig. 5g). Regulator of calcineurin (RCAN) is a calcineurin-interacting protein [32]. The splicing variant RCAN1.4 is directly related to pathological cardiac remodeling, while the other variant, RCAN1.1, usually remains without change. We found that RCAN1.4 was dramatically upregulated in the cKO hearts at both mRNA (Fig. 5g) and protein (Fig. 5h, i) levels, whereas RCAN1.1 was not altered. Consistent with the role of GRP78 in regulation of the UPR, genes of the three signaling pathways were all significantly induced at mRNA (Fig. 5j) and protein levels (Supplementary Fig. S3a-b). Taken together, these data suggest that GRP78 plays an indispensible role in maintaining cardiac homeostasis and ensuring proper cardiac performance.

On the other hand, excessive expression of GRP78 may be equally detrimental to cardiac myocyte survival. To address this, we generated an inducible GRP78 overexpression mouse model by placing GRP78 under the control of a ubiquitous CAG promoter (Supplementary Fig. S4a). A transcriptional/translational stop cassette was inserted between the promoter and GRP78, which was flanked by two loxP sites. When bred to the αMHC-Cre transgenic mouse, Cre causes excision of the stop region and triggers GRP78 expression only in cardiac myocytes (Supplementary Fig. S4b). A total of seven founder lines were identified, none of which showed baseline phenotype (data not shown). We then crossed these individual founders to αMHC-Cre mice. We harvested hearts upon weaning and found that most of the transgenic lines showed significant upregulation of GRP78 (Supplementary Fig. S4c). Among them, the highest expresser, the B11 line, showed approximately 100-fold induction (Supplementary Fig. S4d-e). At weaning, the B11 double transgenic mice manifested severe cardiac dilation, decline of cardiac ejection fraction, and mortality (data not shown). In aggregate, GRP78 plays a pivotal role in cardiac myocyte homeostasis, and too much or too little is not compatible with cardiomyocyte survival and contractile function.

### GRP78 knockdown in vitro in cardiomyocytes leads to increases in cell death

**Fig. 6:**
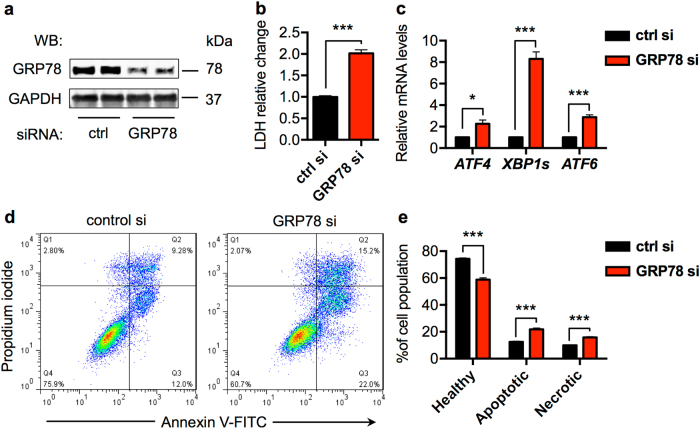
GRP78 knockdown in vitro in cardiomyocytes leads to increases in cell death. **a** GRP78 knockdown by siRNA in cultured primary ventricular myocytes. GAPDH was used as a loading control. **b** Knockdown of GRP78 in NRVMs increased cell death as assayed by lactate dehydrogenase (LDH) release to the culture medium. *N* = 4. **c** GRP78 knockdown in NRVMs led to induction of ER stress markers as examined by qPCR. *N* = 6–7. **d** Flow cytometry using Annexin V and propidium iodide staining in NRVMs showed increases in apoptosis by GRP78 knockdown. **e** Quantification of **d** showed significant increases in both apoptosis and necrosis in NRVMs after GRP78 knockdown. *N* = 4. *p < 0.05; ***p < 0.001

We then used cultured NRVMs to delineate the underlying mechanism of cell death in the absence of GRP78. NRVMs were transfected by specific small interfering RNA (siRNA) against GRP78. GRP78 was significantly reduced by >50% (Fig. 6a and Supplementary Figure S5a). Knockdown of GRP78 caused strong increases in cell death as assessed by lactate dehydrogenase (LDH) release (Fig. 6b). Consistently, the UPR markers were significantly upregulated (Fig. 6c and Supplementary Fig. S3c-d).

We next took advantage of flow cytometry to determine the type of cell death. After siRNA-mediated knockdown, NRVMs were stained with Annexin V and propidium iodide (PI) (Fig. 6d). We found that both apoptosis and necrosis were significantly elevated by GRP78 knockdown (Fig. 6e). Interestingly, the other independent siRNA caused less profound knockdown of GRP78 (Supplementary Fig. S5a-b), which was associated with fewer cell death (Supplementary Fig. S5c), lower induction of the UPR markers (Supplementary Fig. S5d), and less apoptosis (Supplementary Fig. S5e-f). These results highlight the dependence of cardiac myocyte survival on GRP78 expression levels.

### GRP78 deficiency suppresses AKT signaling

**Fig. 7:**
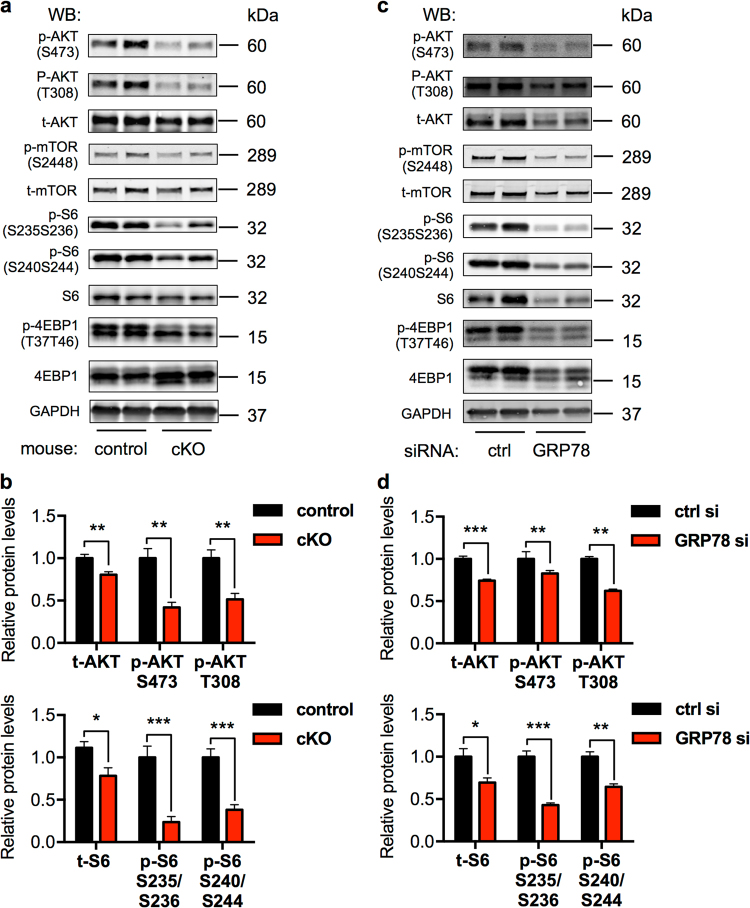
GRP78 deficiency suppresses AKT signaling. **a** Cardiac myocyte-specific knockout of GRP78 in adult mice led to decreases in AKT and mTOR signaling. GAPDH was used as a loading control. **b** Quantification to GAPDH of **a** showed significant suppression in AKT phosphorylation and mTOR activation. *N* = 6. **c** GRP78 knockdown by siRNA in NRVMs led to inhibition of AKT signaling and the mTOR pathway. GAPDH was used as a loading control. **d** Relative quantification to GAPDH of **c** showed significant reduction of AKT and mTOR signaling by GRP78 knockdown in NRVMs. *N* = 4–7. *p < 0.05; **p < 0.01; ***p < 0.001

AKT is a pro-survival signaling nexus downstream of multiple growth factors and hormones. Accumulating studies have shown that GRP78 may directly stimulate AKT [33]. Here we found that cardiac myocyte-specific elimination of GRP78 led to strong reduction in either total AKT level or AKT phosphorylation (S473 and T308) (Fig. 7a, b). Additionally, relative phosphorylation over total AKT was significantly suppressed (Supplementary Fig. S6a). Consistently, the mammalian target of rapamycin (mTOR) pathway, downstream of AKT, was similarly impaired by GRP78 deficiency (Fig. 7a, b and Supplementary Fig. S6a). However, autophagy was not altered (Supplementary Fig. S3a-b).

To examine whether inhibition of AKT signaling was a cell-autonomous phenomenon, we reduced GRP78 by siRNA in NRVMs. We found that both AKT protein level and phosphorylation were significantly attenuated in knockdown cells (Fig. 7c, d), which was accompanied by decreases in mTOR signaling (Fig. 7c, d). Similarly, relative phosphorylation levels were strongly inhibited (Supplementary Fig. S6b), without significant changes in autophagy gene expression (Supplementary Fig. S3c-d). We went on to examine the underlying mechanism by which GRP78 regulated AKT. Previous studies have shown that GRP78 may migrate to the plasma membrane and stimulate phosphoinositide-3 kinase (PI3K)-AKT signaling [33]. To test this, we conducted a biotinylation assay on cell membrane proteins. We found that GRP78 was localized on myocyte membrane at a considerably high level (Supplementary Figure S7a). Cell membrane-localized GRP78 was significantly diminished upon siRNA-mediated knockdown, correlated with reduced AKT signaling. Importantly, we found that GRP78 and p85, the regulatory subunit of PI3K, were colocalized in cell membrane (Supplementary Figure S7b), which was further corroborated by immunofluorescent staining for GRP78 and PIP3 (Supplementary Fig. S7b). Taken together, these results suggest that deficiency of GRP78 causes suppression of AKT at both in vivo and in vitro levels.

**Fig. 8:**
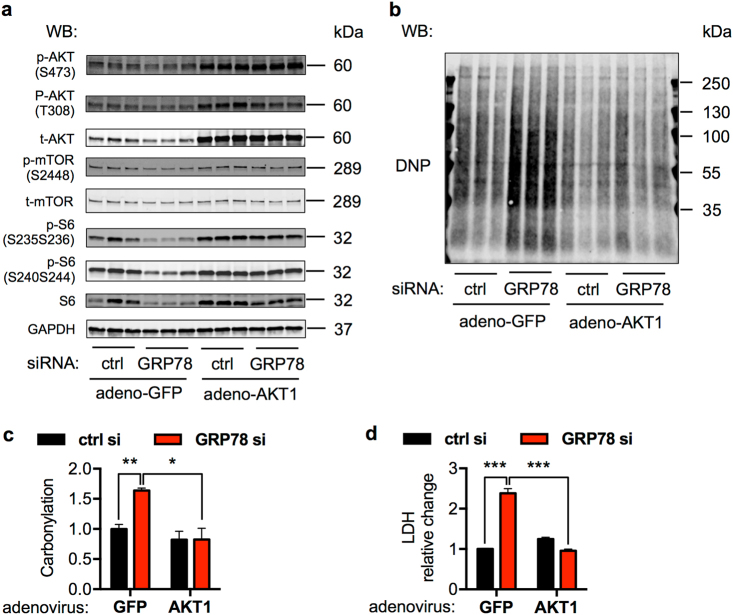
GRP78 knockdown increases ROS production. **a** Overexpression of AKT1 by adenovirus upregulated AKT1 levels and downstream mTOR signaling in NRVMs. Cells were first transfected by siRNA, and then adenovirus expressing either control GFP or a constitutively active mutant of AKT1 was used to infect the NRVMs. GAPDH was used as a loading control. **b** GRP78 knockdown in NRVMs caused increases in protein carbonylation. Activation of AKT1 by adenovirus-mediated overexpression led to decreases in this protein modification. **c** Quantification of **b** showed that protein carbonylation was significantly upregulated in GRP78 knockdown cells, which was attenuated by AKT1 overexpression and activation. *N* = 3. **d** AKT1 activation rescued cell death from GRP78 knockdown. NRVMs were transfected by siRNA against GRP78 and then infected by AKT1-overexpressing adenovirus, followed by a LDH assay to assess cell death. *N* = 3. *p < 0.05; **p < 0.01; ***p < 0.001

Production of reactive oxygen species (ROS) holds a central stage in survival, death, and function of cardiac myocytes [34]. Moreover, accumulating evidence indicates an intricate connection between AKT and ROS production [35]. Here we set out to examine whether ROS played a role in GRP78 deficiency-induced cell death. Protein carbonylation is a type of amino acid side chain modification on lysine, arginine, proline, and threonine. These moieties are stable and readily detectable, which can serve as a biomarker of oxidative protein damage. We first reduced GRP78 expression by siRNA (Fig. 8a) and then subjected cell lysates to the carbonylation assay. We found that knockdown of GRP78 increased protein carbonylation (Fig. 8b, c). Since siRNA-mediated knockdown of GRP78 caused decreases in both total and phosphorylated AKT, we went on to test the possibility of rescuing cardiac cells by overexpressing AKT1. We transfected NRVMs with GRP78 siRNA, followed by adenoviral infection expressing either green fluorescent protein (GFP) or a constitutively active mutant of AKT1 (Fig. 8a). AKT1 overexpression strongly suppressed protein carbonylation (Fig. 8b, c) and prevented GRP78 knockdown-triggered cell death (Fig. 8d). Moreover, pharmacological activation of AKT by SC-79 significantly diminished protein carbonylation (Supplementary Fig. S8a-b), which was accompanied by increases in cell survival (Supplementary Fig. S8c).

We next set to determine the source of ROS upon GRP78 knockdown. Here diphenyleneiodonium (DPI) and MitoTEMPO were used to suppress ROS generation from NADPH oxidase and mitochondria, respectively. The increases in protein carbonylation in GRP78 knockdown cells were significantly suppressed by DPI treatment, whereas MitoTEMPO was not effective (Supplementary Fig. S8d-e). Consistently, DPI led to strong rescue from cell death in GRP78 knockdown NRVMs, suggesting GRP78-AKT regulated ROS is largely mediated by NADPH oxidase (Supplementary Fig. S8f). In aggregate, these results suggest that GRP78 is critical to regulate cellular ROS levels through modulation of the AKT signaling, which is essential to maintain cardiac myocyte homeostasis.

## Discussion

Mounting evidence supports the essential role of GRP78 in UPR regulation, cell survival, and metabolic control [6]. Previous studies have shown that GRP78 is augmented in several forms of heart disease, including pathological cardiac hypertrophy [22], ischemia [23], and ischemia/reperfusion [8]. However, its role in cardiomyocyte physiology remains elusive. Here, we undertook cardiac myocyte-specific gain- and loss-of-function animal models and in vitro tissue culture to characterize the function of GRP78. Deficiency of GRP78 in cardiomyocytes during embryonic development causes severe defects in cardiogenesis and embryos die in utero. We next initiated the deletion of GRP78 in cardiac myocytes from adult mice. Cardiomyocyte death quickly ensues and knockout animals show complete mortality within 3 weeks. On the other hand, transgenic excessive overexpression of GRP78 in the heart leads to severe thinning of cardiac ventricle and deleterious heart failure. Further analysis indicates that GRP78 deficiency greatly suppresses AKT signaling, which may contribute to the elevated apoptosis. Taken together, these results indicate that proper expression of GRP78 is critical for cardiac myocyte survival and heart function, with too much or too little causing detrimental consequences.

GRP78 promoter activity is detected in trophectoderm and inner cell mass at the blastocyst stage [18]. Deficiency of GRP78 leads to peri-implantation lethality and no live embryos exist beyond E3.5. GRP78 expression in the heart is largely driven by transcriptional regulation since a β-galactosidase reporter animal model [36] governed by GRP78 promoter faithfully recapitulates endogenous GRP78 expression at both the mRNA [36] and protein [37] levels. Importantly, the heart is of the highest expression of GRP78 at E11.5, compared with brain and somites. Consistently, we found that deficiency of GRP78 only in cardiomyocytes shows cardiac defects at E12.5 and all embryos die before birth.

While much of the GRP78 action may be mediated by the chaperone function, emerging evidence suggests that GRP78 has additional roles above and beyond UPR regulation [33]. Early studies show that thapsigargin induction of GRP78 triggers partial translocation to the plasma membrane [38]. Later, cell membrane localization of GRP78 has been identified in human prostate cancer cells [39, 40], breast cancer cells [41], human glioblastoma multiforme cells [42], murine macrophages [43], etc. In addition, overexpression of GRP78 in HEK293 cells stimulates cell membrane relocation [44], suggesting that upregulation of GRP78 in cancer cells may be the primary reason of membrane migration.

Plasma membrane-localized GRP78 plays an important role in promoting cell survival and proliferation. Misra et al. showed that cell membrane GRP78 may serve as a receptor for activated α2-macroglobulin, which participates in calcium accumulation [39], AKT activation, and apoptosis suppression [45]. Along these lines, a monoclonal antibody targeting cell surface GRP78 causes reduction in breast cancer cell proliferation, halted AKT activation, and impaired tumor growth [46]. At the mechanistic level, cell surface-localized GRP78 may directly interact with PI3K and stimulate PIP3 production, which in turn activates AKT [47]. Importantly, an insertion mutation that does not affect cell membrane relocation of GRP78 but disrupts PI3K binding is defective in PI3K activation and AKT phosphorylation [47]. Our results here are consistent with a pro-surviving role of GRP78 in cardiac myocytes. Taken together, these studies indicate that cell surface-localized GRP78 may directly stimulate AKT, which can promote survival and proliferation in multiple cell types under various conditions.

Despite that our work points to an essential role of GRP78 in cardiomyocyte survival, several questions remain. We have shown that membrane-localized GRP78 is critical for AKT phosphorylation. However, other mechanisms may exist to account for the reduction of AKT activity upon GRP78 deficiency. Indeed, Avery et al. showed that UPR induction by pharmacological inducers suppresses AKT, which is mediated by augmentation of Tribbles 3 (TRB3) [48]. Since the decrease in GRP78 by siRNA leads to upregulation of the UPR, TRB3 may therefore be stimulated to inhibit AKT. Future work is required to elucidate potential mechanisms beyond membrane translocation of GRP78. Moreover, additional role of GRP78 as a protein-folding chaperone remains to be explored. Deficiency of GRP78 may adversely affect cell survival by creating defects in key secretory or transmembrane proteins in the heart.

Here our work at both in vivo and in vitro levels strongly supports that GRP78 is indispensible for physiological function and survival of cardiac myocytes. Embryonic deficiency of GRP78 in cardiomyocytes causes developmental defects in the heart and lethality before birth. Moreover, acute deletion of GRP78 in adult animals leads to cardiomyocyte loss, severe heart failure, and early death. Our results therefore highlight the critical role of GRP78 in preventing cardiac cell death, maintaining contractile function and systolic performance, and survival.

## Materials and methods

### Animals

All mice were in the C57BL/6 background. Animals were maintained on a 12 h dark/light cycle (6 AM to 6 PM) and housed in a barrier facility in groups of <4 with unlimited access to water and chow (2916, Teklad). The Institutional Animal Care and the Use Committee of University of Texas Southwestern Medical Center has approved all animal experiments.

To engineer cardiac-specific knockout of GRP78, the GRP78^fl/fl^ mouse [18, 19] was crossed with cardiomyocyte-specific Cre (αMHC-Cre) mouse model. Since the αMHC promoter is active in embryonic stage, GRP78 was eliminated in cardiomyocytes during early development. We bred GRP78^fl/fl^ with αMHC-Cre;GRP78^fl/+^ to generate αMHC-Cre;GRP78^fl/fl^ animals. To create the inducible and cardiomyocyte-specific knockout model, we crossed the GRP78^fl/fl^ mouse with the αMHC-MCM animal model. Nuclear translation and activation of MCM was achieved by 5 consecutive days of tamoxifen (T5648, Sigma) injection (20 mg/kg body weight in peanut oil). Animal mortality was observed daily. All primers are provided in Supplementary Table S1.

### Echocardiography

Cardiac function was monitored by echocardiography on conscious, gently restrained mice with a Vevo 2100 system (MS400C probe, VisualSonics) [8]. M-mode images were captured and analyzed. LVID;diastole and LVID;systole were quantified, and fractional shortening (FS%) and ejection fraction (EF%) were calculated. All measurements were conducted at the level of papillary muscles.

### Transmission electron microscopy

The hearts were harvested at days 7 and 14 post the initiation of tamoxifen injection. Cardiac tissues were cut into small cubes of 1 mm dimension and fixed in freshly prepared 2.5% gluteraldehyde in 0.1 M sodium cacodylate buffer for 2 h. Samples were then fixed in 1% osmium tetroxide containing 0.8% potassium ferricyanide for 1.5 h in the dark. After washing for five times in water, cardiac samples were stored in refrigerator overnight. Samples were then enbloc stained in 4% uranyl acetate in methanol for 2 h in the dark and dehydrated through a graded ethanol series (50%, 70%—two times, 85%, 95%—two times, and 100%—4 times) for 8 min each step. Samples were then placed in propylene oxide for 10 min and infiltrated with propylene oxide/EPON 812 (2:1) for 2 h on a rotator and then propylene oxide/EPON 812 (1:2) for 2 h. The samples were left under fume hood overnight. In the following day, samples were placed into 100% EPON 812 for 2 h two times on rotator and then embedded in EPON 812 and polymerized in a 60 °C oven. Tissues were sectioned with a Leica UC7 ultramicrotome with a Diatome diamond knife at 80 nm thick. Sections were stained with 2% aqueous uranyl acetate for 14 min, followed by lead citrate for 4 min. Samples were observed in a Tecnia G2 Spirit TEM at 120 kv.

### NRVM isolation and culture

Ventricles from 1- to 2-day-old Sprague-Dawley rats were harvested and subjected to cardiomyocyte isolation using a neonatal rat/mouse Cardiomyocyte Isolation Kit (NC-6031, Cellutron). Fibroblasts were removed by preplating for 2 h. Cardiomyocytes were then plated in 6-well plates at a density of 1250 cells/mm^2^ in plating medium (Dulbecco’s modified Eagle’s medium/M199, containing 5% fetal bovine serum, 10% horse serum, and 100 μM bromodeoxyuridine).

### Pulse-chase labeling experiments in NRVMs

Cells were cultured in 10-cm plates. Radioactive ^35^S-methionine/cysteine (0.3 mCi/mL, Perkin Elmer) was incubated in culture medium for 15 min. Cells were then washed and replenished with fresh medium containing 300 μM cycloheximide. Culture medium was collected every 30 min for 3 h. Half of the aliquot was used for PNGase F (P0704S, NEB) treatment to remove *N*-glycan. Samples were then loaded to SDS-PAGE gels and subjected to autoradiography.

### Histology

Neonatal embryos and adult (12 week old) hearts were harvested and immediately fixed in 4% paraformaldehyde for 48 h. Paraffin-embedded tissues were used for sectioning (5 μm). Hematoxylin and eosin staining and Masson’s Trichrome staining were performed by the Molecular Pathology Core. TUNEL was conducted according to the manufacturer’s recommendations (DeadEnd fluorometric TUNEL system, Promega). For immunofluorescence staining, sections were first deparaffinized and rehydrated. Antigens were retrieved with antigen retrieval citra solution (HK086-9K, BioGenex) and non-specific binding was blocked by normal goat serum. Primary antibodies were used to incubate overnight. Sections were then stained with secondary antibodies for 1 h at room temperature. After six washes, sections were counterstained by ProLong Gold antifade reagent with 4,6-diamidino-2-phenylindole (P36935, Thermo), and images were captured by a fluorescent microscope. The following antibodies were used: anti-α-actinin (ab68167, Abcam), anti-GRP78 (610979, BD Biosciences), goat anti-rabbit secondary antibodies Alexa Fluor 488 (A-11008, Thermo), and goat anti-mouse secondary antibodies Alexa Fluor 568 (A-11011, Thermo). For wheat germ agglutinin (WGA) staining, after deparaffinization and rehydration, heart sections were blocked by 5% normal goat serum. Alexa Fluor 594-conjugated WGA was used to incubate with the slides (W11262, 10 μg/mL, Thermo) for 1 h. After mounting with ProLong Gold antifade, sections were imaged with a fluorescent microscope (Leica).

### Adenovirus infection

Prepackaged adenovirus expressing HA-tagged constitutively active AKT1 was obtained from Vector Biolabs (1020). NRVMs were first transfected by GRP78 siRNA. Adenovirus was used to infect NRVMs for 6 h (multiplicity of infection: 5–10). GFP-expressing adenovirus was used as a control. Cell death was then determined by a LDH release assay. We routinely obtained >70% infection rate.

### Immunoblotting analysis

NRVMs were lysed in RIPA (radioimmunoprecipitation assay buffer) supplemented with protease and phosphatase inhibitors (A32959, Thermo). After a cycle of freezing/thawing, lysates were cleared by centrifugation at 4 °C (14,000 RPM, 10 min). Supernatant was used for protein quantification with a BCA assay (23225, Thermo). For the heart, cardiac tissues of 20 mg were homogenized by dounce homogenizer in 500 μL RIPA, followed by centrifugation and concentration determination. Equal amount of total protein was loaded onto a Criterion gel (Bio-Rad). After transferring to the nitrocellulose membrane, immunoblotting was done by incubating with primary antibodies overnight, followed by secondary antibodies for 1 h. Immunoblots were then visualized by an Odyssey scanner (Li-Cor) and quantified with the ImageStudio software (Li-Cor). The following antibodies were used: GRP78 (610979, BD Biosciences), GAPDH (glyceraldehyde 3-phosphate dehydrogenase; 10R-G109A, Fitzgerald), p-AKT (T308) (13038, Cell Signaling), p-AKT (S473) (4060, Cell Signaling), total-AKT (9272, Cell Signaling), RCAN1 (D6694, Sigma), mTOR (4517, Cell Signaling), p-mTOR (2971, Cell Signaling), S6 (2317, Cell Signaling), p-S6 S235S236 (4858, Cell Signaling), p-S6 S240S244 (5364, Cell Signaling), 4EBP1 (9644, Cell Signaling), p-4EBP1 (2855, Cell Signaling), goat anti-rabbit secondary antibody 800 CW (925–32211, Li-Cor), and goat anti-mouse secondary antibody Alexa Fluor 680 (A21057, Thermo).

For the protein carbonylation assay, NRVM lysates were prepared in RIPA and quantified by a BCA assay (Thermo). Each sample was split into two equal portions, one for DNPH (2,4-dinitrophenylhydrazine, Sigma) and the other for solvent trifluoroacetic acid treatments. Reaction was then neutralized with Tris solution (2 M), and samples were loaded onto a Criterion gel for separation and detection with anti-DNP antibodies (MAB2223, Millipore).

### RNA isolation and PCR

Total RNA from cardiac tissues and NRVMs was isolated using a Total RNA Fatty and Fibrous Tissue Kit (Bio-Rad) and a Quick-RNA MicroPrep Kit (Zymo Research), respectively. Approximately 250 ng total RNA from each sample was removed for reverse transcription using the iScript Supermix (Bio-Rad). After dilution in distilled water (1:10), cDNA (2 μL/sample) was used for qPCR on a LightCycler 480 (Roche). Relative mRNA levels were calculated using the 2^−ΔΔCt^ method with 18s RNA as internal control.

### Flow cytometry

NRVMs were trypsinized, harvested, and resuspended in 100 μL phosphate-buffered saline. Annexin V-fluorescein isothiocyanate and PI staining solution (Biotool) was added. The cells were then subjected to flow cytometry using FAScan, and results were analyzed with Flowjo.

### Statistics

Statistical analysis was performed using the GraphPad Prism software. All data are expressed as mean ± S.E.M. The Student’s *t*-test (two-tailed) was conducted to compare difference between two groups. In addition, one-way or two-way analysis of variance was used for multiple groups. Tukey test was then performed to evaluate differences between the mean values of paired groups. For survival studies, the Gehan–Breslow–Wilcoxon test was conducted. *p* < 0.05 was considered statistically significant.

## Electronic supplementary material

Supplementary materials
