# Supplementary material for: Glucose-regulated protein 78 is essential for cardiac myocyte survival
Source: Cell Death Differ. 2018 Apr 17;25(12):2181–94. doi: 10.1038/s41418-018-0109-4 (PMC6261960; doi:10.1038/s41418-018-0109-4)
Supplement: Supplementary file 1 — Supplementary materials [file 41418_2018_109_MOESM1_ESM.pdf]

Supplementary Materials

Glucose-regulated protein 78 is essential for cardiac myocyte survival

Xiaoding Wang[1,2], Xukun Bi[2,3], Guangyu Zhang[2,4], Yingfeng Deng[5], Xiang Luo[2], Lin Xu[1], Philipp E. Scherer[5], Anwarul Ferdous[2], Guosheng Fu[3], Thomas G. Gillette[2], Amy S. Lee[6], Xuejun Jiang[1,*] and Zhao V. Wang[2,*]

[1]Department of Cardiology, Renmin Hospital of Wuhan University, Wuhan, Hubei, China

[2]Division of Cardiology, Department of Internal Medicine, University of Texas Southwestern Medical Center, Dallas, TX, USA

[3]Department of Cardiology, Biomedical Research (Therapy) Center, Sir Run Run Shaw Hospital, School of Medicine, Zhejiang University, Hangzhou, Zhejiang, China

[4]Department of Cardiology, Zhongnan Hospital of Wuhan University, Wuhan, Hubei, China

[5]Touchstone Diabetes Center, Department of Internal Medicine, University of Texas Southwestern Medical Center, Dallas, TX, USA

[6]Department of Biochemistry and Molecular Medicine, USC Norris Comprehensive Cancer Center, Keck School of Medicine, University of Southern California, Los Angeles, CA, USA

Correspondence:

*Xuejun Jiang, M.D., Ph.D.

Department of Cardiology, Renmin Hospital of Wuhan University, 238 Jiefang Road, Wuhan, Hubei, China, 430060.

Tel: 86-27-88041911

Email: xjjiang@whu.edu.cn

*Zhao V. Wang, Ph.D.

Division of Cardiology, Department of Internal Medicine, University of Texas Southwestern Medical Center, 5323 Harry Hines Blvd., Dallas, TX, USA, 75390-8573.

Tel: 1-214-648-6686; Fax: 1-214-648-1450

Email: zhao.wang@utsouthwestern.edu

Supplementary Figure S1.

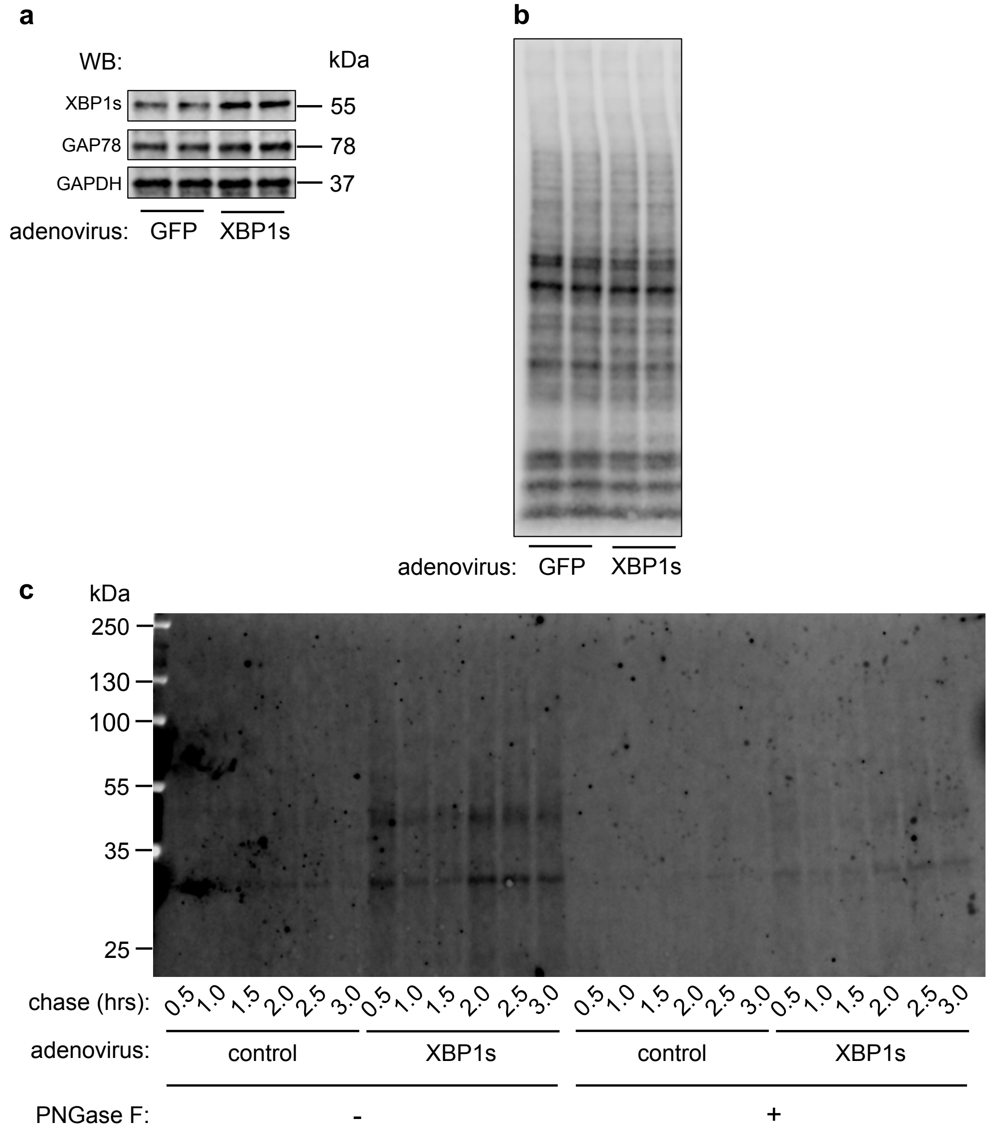

Supplementary Figure S1. Overexpression of XBP1s augments the secretory capacity of NRVMs.

(a) NRVMs were infected by adenovirus expressing either GFP or XBP1s. Cell lysates were extracted for immunoblotting for XBP1s. GRP78 as a target of XBP1s was significantly elevated by XBP1s overexpression. GAPDH was used as a loading control. **(b)** Overexpression of XBP1s in NRVMs did not alter total protein levels. Cell lysates were loaded to a Criterion gel for separation. After transferring to the nitrocellulose membrane, proteins were stained by the Total Protein Staining reagent (Li-Cor) and scanned in a Li-Cor fluorescent imager. No detectable difference was noticed between GFP and XBP1s groups. **(c)** The secretory capacity of NRVMs was enhanced by XBP1s overexpression. A pulse-chase experiment was conducted after nascent proteins were labeled by Click-iT AHA, an amino acid analog. Culture medium was removed every 30 min and subjected to Click-iT detection using the TAMRA detection reagent. After PNGase F treatment, the secretory proteins were resolved in a Criterion gel and used for fluorescent detection with the Li-Cor scanner Fc. Note that XBP1s overexpression led to increases in secretory proteins and PNGase F treatment accelerated protein migration.

Supplementary Figure S2.

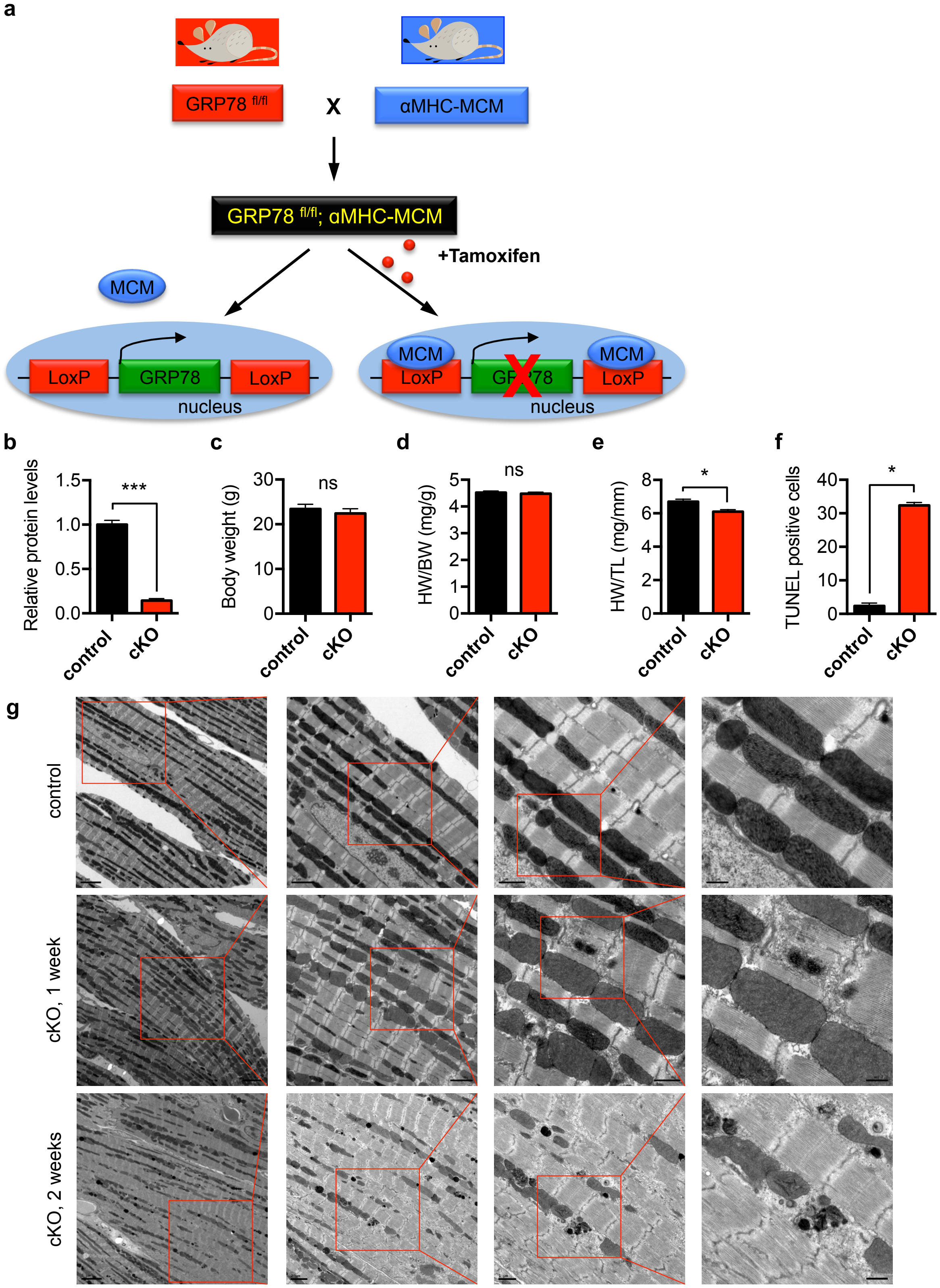

Supplementary Figure S2. The inducible GRP78 knockout mouse model.

(a) To achieve inducible and cardiomyocyte-specific knockout of GRP78, the GRP78$^{fl/fl}$ mouse was crossed to cardiomyocyte-restricted MerCreMer animal (αMHC-MCM). In the compound mouse model GRP78$^{fl/fl}$;αMHC-MCM, MCM was sequestered in the cytosol and GRP78 genomic locus was intact. Upon administration of tamoxifen, MCM was liberated and translocated to the nucleus, where Cre triggered excision and elimination of GRP78. **(b)** Cardiomyocyte-specific knockout of GRP78. Quantification of GRP78 showed significant reduction in GRP78 at protein level in the cKO mice compared to controls. N = 6. **(c)** GRP78 cKO in the heart did not affect animal body weight. N = 7-9. **(d)** The ratios of heart weight/body weight (HW/BW) did not differ between control and cKO mice. N = 7-9. **(e)** Heart weight/tibia length (HW/TL) showed a moderate but significant reduction in the cKO mice. N = 5. **(f)** GRP78 knockout in the heart increased cardiac myocyte cell death as assessed by TUNEL staining. Positive cells across the whole heart section were quantified and compared between control and cKO groups. N =3. **(g)** Electron microscopic analysis of the hearts at ultrastructure level. Note that the cKO heart showed severe disarray of sarcomeres and disruption of mitochondrial cristae at 1 week post tamoxifen exposure, which were exacerbated at 2 weeks after tamoxifen injection. *, $p < 0.05$; ***, $p < 0.001$; ns, not significant.

Supplementary Figure S3.

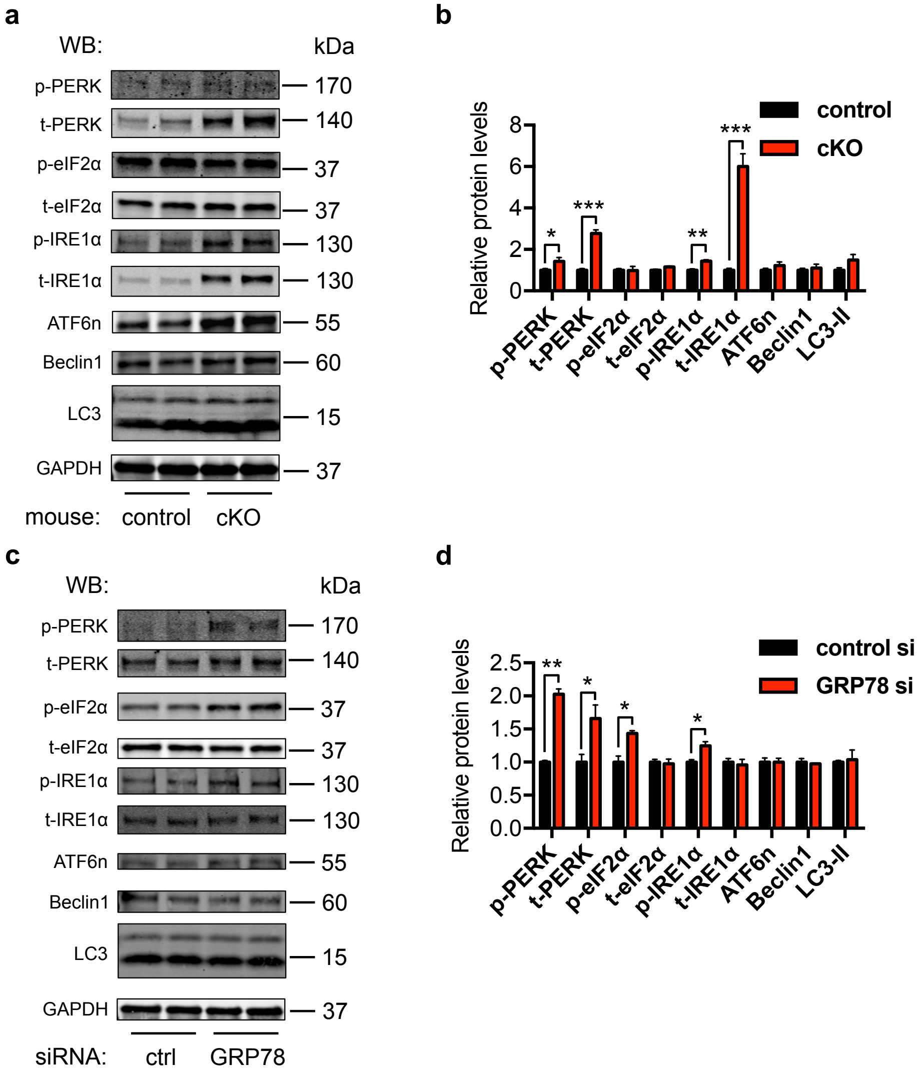

Supplementary Figure S3. GRP78 deficiency leads to elevation of the UPR related proteins.

(a) Cardiac tissues from control and GRP78 cKO mice were subjected to immunoblotting to detect changes in UPR transducers and autophagy related proteins. GAPDH was used as a loading control. **(b)** Quantification of a) showed significant elevation in several markers of the UPR. Note that Beclin1 and LC3-II did not alter, indicating autophagy may not change in cKO hearts. N = 3-6. **(c)** GRP78 was reduced by siRNA-mediated knockdown and cell lysates were used for Western blotting. GAPDH was used as loading control. **(d)** Quantification of c) showed significant increases in multiple transducers of the UPR. However, Beclin1 and LC3-II did not show significant changes. N = 3-4. * < 0.05; **, p < 0.01; ***, p < 0.001.

Supplementary Figure S4.

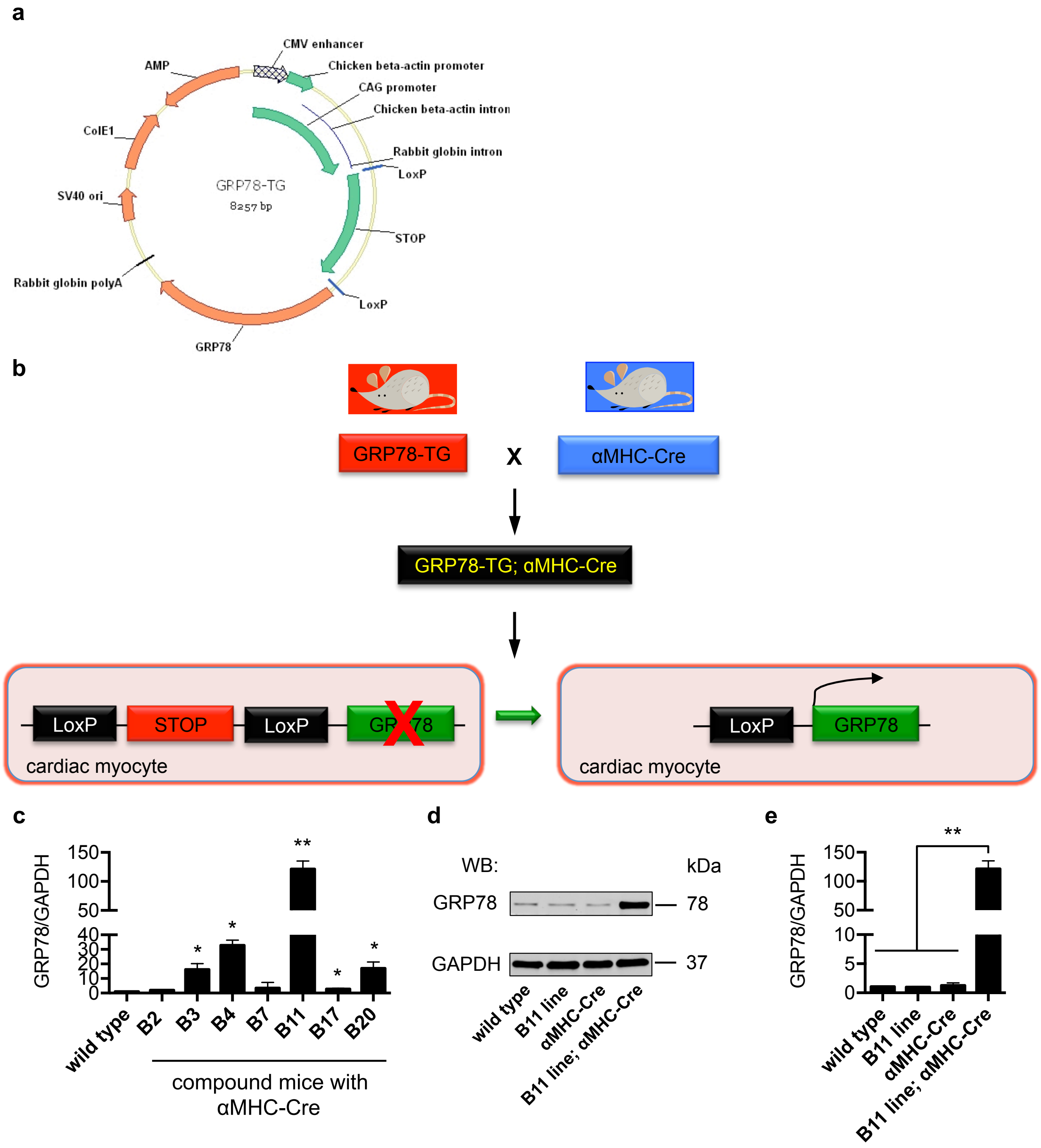

Supplementary Figure S4. The inducible GRP78 transgenic mouse model.

(a) The GRP78 transgenic construct. Mouse GRP78 gene was placed under the control of the CAG promoter. A transcriptional/translational stop cassette was inserted between CAG and GRP78, which was flanked by two loxP sites. **(b)** In the double transgenic GRP78-TG;αMHC-Cre mouse model, the stop region is cleaved and removed by Cre, only in cardiac myocytes. GRP78 overexpression therefore ensues. **(c)** All GRP78-TG founder lines were bred to the αMHC-Cre mouse model individually. We harvested the hearts upon weaning and analyzed GRP78 expression by immunoblotting. Founder lines B3, B4, B11, B17 and B20 showed significant upregulation compared to wild type controls. N = 3-5. **(d)** In the highest expression

line, B11, GRP78 was only induced in the double transgenic hearts compared to the other three control genotypes. **(e)** GRP78 was augmented by approximately 100-fold in the line B11. N = 3-7. *, $p < 0.05$; **, $p < 0.01$.

Supplementary Figure S5.

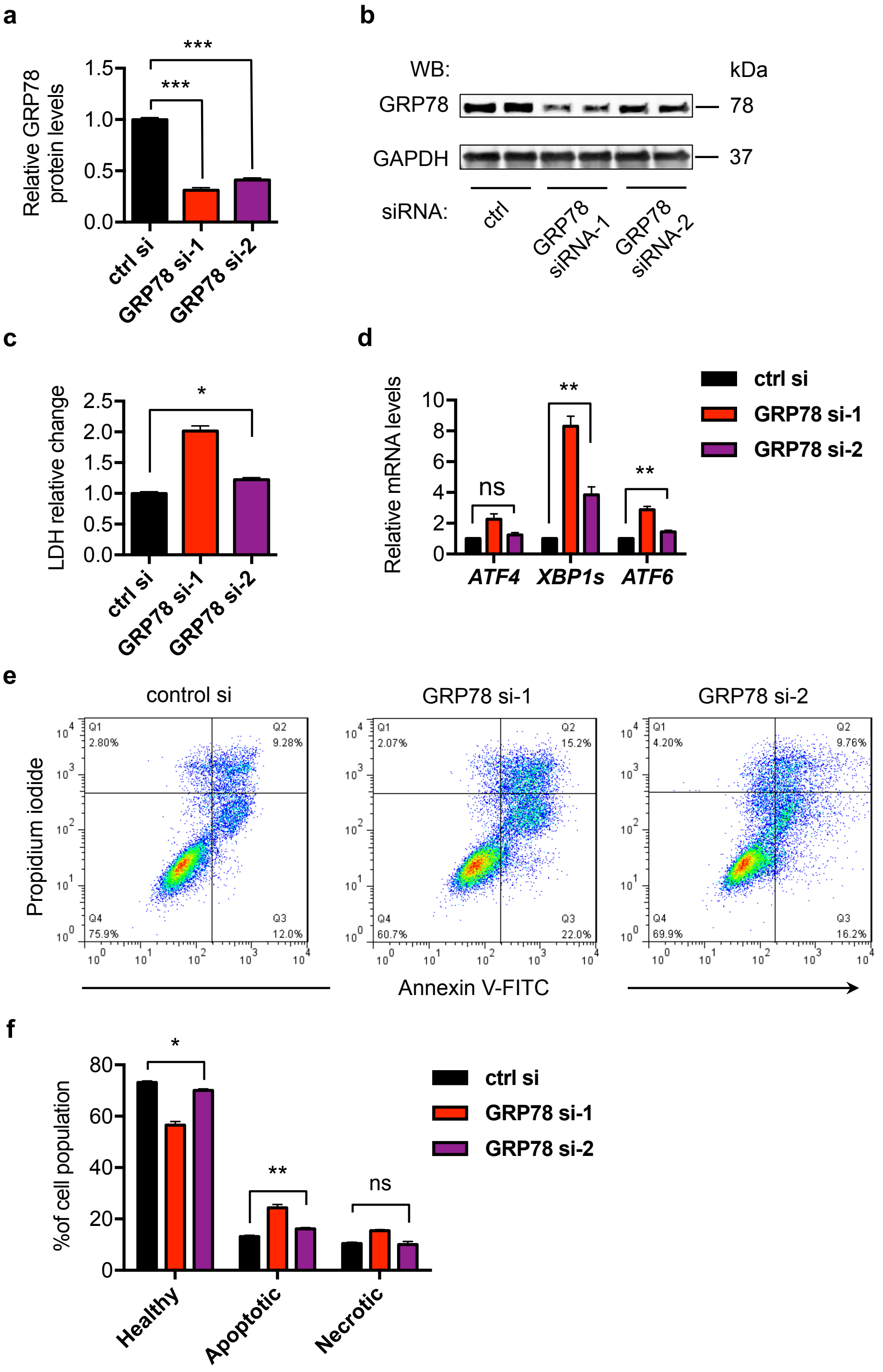

Supplementary Figure S5. GRP78 knockdown *in vitro* in cardiomyocyte culture.

(a) Quantification showed GRP78 was significantly reduced at protein level after specific siRNA transfection compared to control siRNA. Note that GRP78 si-1 was more efficient in reducing GRP78 expression compared to GRP78 si-2. N =3-6. **(b)** A representative immunoblot of GRP78 after siRNA knockdown in NRVMs. GAPDH was used as a loading control. **(c)** GRP78

knockdown by GRP78 si-2 caused significant cell death, albeit less than GRP78 si-1. N = 4. **(d)** Both siRNAs led to induction of the UPR markers as assessed by qPCR. N = 6-7. **(e)** Flow cytometry showed that siRNA-mediated knockdown of GRP78 increased cell death. **(f)** GRP78 si-2 led to decreases in cell viability and increases in apoptosis. The degree of cell death was less than si-1, which was consistent with less profound knockdown by si-2. N = 3-6. *, $p < 0.05$; **, $p < 0.01$; ***, $p < 0.001$; ns, not significant.

Supplementary Figure S6.

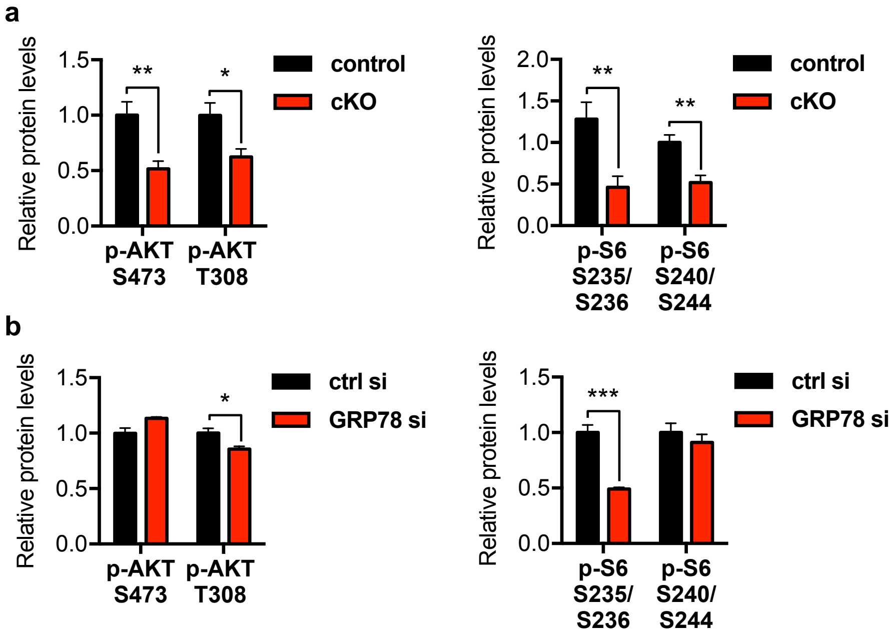

Supplementary Figure S6. GRP78 deficiency inhibits AKT signaling.

(a) Relative quantification to total AKT (left) and total S6 (right) showed a significant decrease in phosphorylation of AKT and S6, respectively in the cKO heart. N = 4-6. **(b)** Relative quantification to total AKT (left) and total S6 (right) showed that phosphorylation of AKT (T308) and S6 (S235/S236) was strongly suppressed in NRVMs after GRP78 knockdown. N = 3-5. *, $p < 0.05$; **, $p < 0.01$; ***, $p < 0.001$.

Supplementary Figure S7.

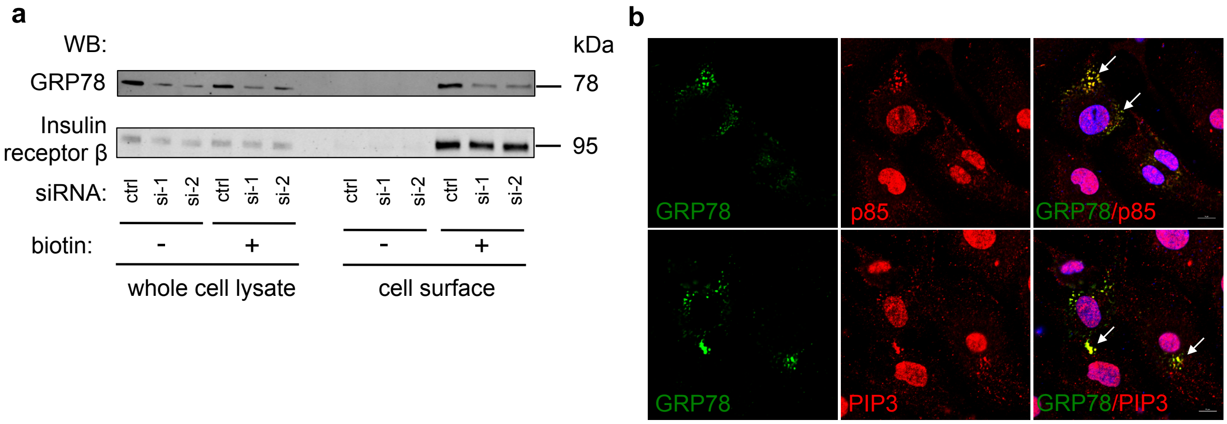

Supplementary Figure S7. GRP78 interacts with PI3K in NRVMs.

(a) NRVMs were transfected with control siRNA or siRNA against GRP78. EZ-link Sulfo-NHS-LC-Biotin was used to label cell surface proteins. After isolation with neutravidin, Western blotting was performed to detect cell surface-localized GRP78. Insulin receptor β was used as a positive control. Cell surface GRP78 was diminished upon siRNA-mediated knockdown. **(b)** Cell surface associated GRP78 was colocalized with p85 and PIP3. NRVMs were stained for GRP78 without cell permeabilization at 4°C, followed by saponin treatment and incubation of p85 antibodies. Arrows indicate colocalization of GRP78 and p85. Co-staining for GRP78 and PIP3 was similarly conducted. Scale bar: 10 μm.

Supplementary Figure S8.

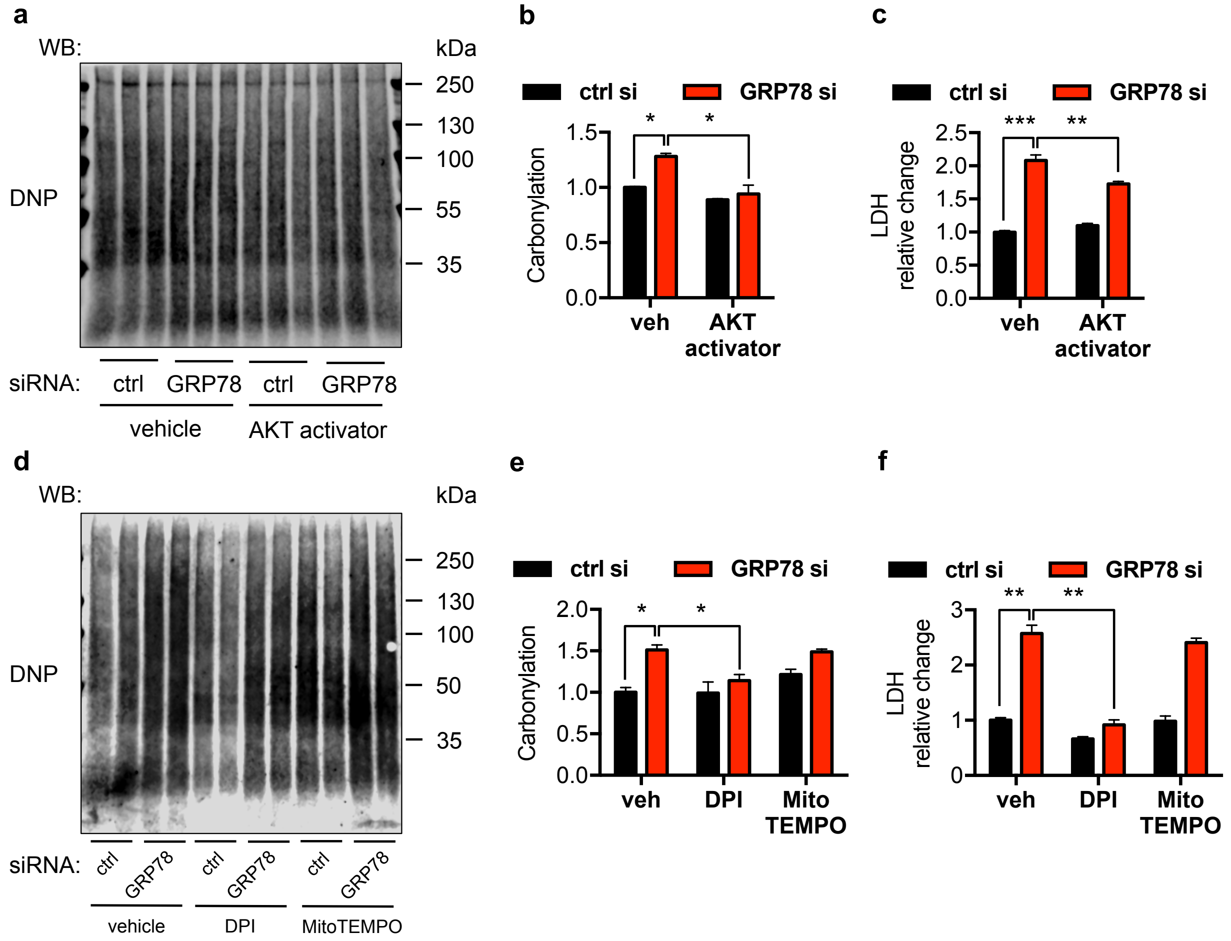

Supplementary Figure S8. GRP78-AKT axis regulates ROS production in NRVMs.

(a) GRP78 knockdown in NRVMs caused increases in protein carbonylation. Activation of AKT by specific activator SC-79 led to decreases in this post-translational protein modification. **(b)** Quantification of a) showed that protein carbonylation was significantly upregulated in GRP78 knockdown cells, which was attenuated by AKT activation. N = 3. **(c)** AKT activation rescued cell death from GRP78 knockdown. siRNA against GRP78 was first transfected and NRVMs were treated by AKT activator SC-79, followed by LDH assay to assess cell death. N = 3-9. **(d)** NADPH oxidase inhibition by DPI decreased protein carbonylation, whereas MitoTEMPO did not significantly alter GRP78 knockdown-mediated ROS generation. NRVMs were first transfected by control or GRP78 siRNA. NADPH oxidase inhibitor DPI (100 nM) or mitochondrial ROS inhibitor MitoTEMPO (10 μM) was supplemented in the culture medium. Protein carbonylation assay was then conducted. **(e)** Quantification of d) showed DPI treatment significantly diminished GRP78 deficiency-induced ROS production. N = 3. **(f)** NADPH oxidase inhibition by DPI strongly prevented GRP78 knockdown-induced cell death as assayed by LDH release. N = 5-6. *, $p < 0.05$; **, $p < 0.01$; ***, $p < 0.001$.

Supplementary Table S1.

Gene	Species	Primer sequence	Test
GRP78	mouse	GCACGGTTGTCCTTCTAGGT<br>CCTCTCTCTTATCCAGGCCA	GRP78$^{fl/fl}$ genotyping
Cre		GATTTCGACCAGGTTCGTTC<br>GCTAACCAGCGTTTTCGTTC	genotyping
MCM		ACCTGCTCCTGGAGATGTTG<br>CATCACTCGTTGCATCGACC	genotyping
GRP78	mouse	CCTCTCTGGTGATCAGGATA<br>CGTGGAGAAGATCTGAGACT	qPCR/ GRP78-TG genotyping
ATF4	mouse	CCACCAGACAATCTGCCTTC<br>CTAGCTCCTTACACTCGCCA	qPCR
ATF6	mouse	GCCGACTGTGGTTCAACTTC<br>TCCTCAGCACAGCGATATCC	qPCR
XBP1s	mouse/rat	GGTCTGCTGAGTCCGCAGCAGG<br>GAAAGGGAGGCTGGTAAGGAAC	qPCR
aMHC	mouse	CGGAACAAGACAACCTCAAT<br>TGGCAATGATTTCATCCAGC	qPCR
BNP	mouse	CATGGATCTCCTGAAGGTGC<br>CCTTCAAGAGCTGTCTCTGG	qPCR
RCAN1.1	mouse/rat	GACCCGCGCGTGTTC<br>TGTCATATGTTCTGAAGAGGGAATC	qPCR
RCAN1.4	mouse/rat	CCCGTGAAAAAGCAGAATGC<br>TCCTTGTCATATGTTCTGAAGAGGG	qPCR
18s	mouse	AGGGTTCGATTCCGGAGAGG<br>CAACTTTAATATACGCTATTGG	qPCR
ATF4	rat	ACCAGACAGTCTGCCTTCTC<br>CTCTTCTTCTGGCGGTACCT	qPCR
ATF6	rat	TCTGCTCAGCCAGTACTTGC<br>CATACGCTGCTGTCTCCTCA	qPCR
18s	rat	AAACGGCTACCACATCCAAG<br>CCTCCAATGGATCCTCGTTA	qPCR
GRP78	rat	CTTCTCAGCATCAAGCGAGG<br>GTAGATCCGCCAACCAGAAC	qPCR

Supplementary materials and methods

Pulse-chase labeling experiments using Click-iT AHA in NRVMs. Cardiac cells were infected by adenovirus expressing either GFP or XBP1s for 24 hrs. NRVMs were then harvested for immunoblotting to verify XBP1s expression. In addition, total protein detection was conducted by using the Revert Total Protein Staining reagent (926-11016, Li-Cor), followed by scanning with a Li-Cor Fc imager. To pulse label secretory proteins, NRVMs were first starved for 30 min with methionine/cysteine-free DMEM (21013024, Thermo). Click-it AHA (C10102, Thermo, L-Azidohomoalanine) was added at 50 μM for 1 hr. After 3 washes, cells were replenished with DMEM containing protein translational inhibitor cycloheximide (300 μM) and culture medium was collected every 30 min for 3 hrs to chase newly synthesized secretory proteins. Click-iT Tetramethylrhodamine (TAMRA) detection was then conducted (C33370, Thermo). Next, each sample was split to two parts, one for PNGase F treatment and other one for treatment with the same condition without the PNGase F enzyme. The Click-iT labeled secretory proteins were then resolved in a Criterion gel (Bio-Rad) and visualized by a Li-Cor Fc scanner at 600 nm.

Immunoblotting. NRVMs or cardiac tissues were lysed in RIPA buffer and subjected to Western blotting. The following antibodies were used: p-PERK (3179, Cell Signaling), t-PERK (3192, Cell Signaling), p-eIF2α (PA5-37800, Thermo), t-eIF2α (AHO1182, Thermo), p-IRE1α (NB100-2323, Novus Biologicals), t-IRE1α (3294, Cell Signaling), ATF6 (73-500-EX, Cosmo Bio.), Beclin1 (3738, Cell Signaling), XBP1 (SC-7160, Santa Cruz Biotechnology), and insulin receptor β (SC-711, Santa Cruz Biotechnology).

Cardiac specific GRP78 transgenic mouse model. To generate tissue-specific GRP78 overexpression mouse model, GRP78 open reading frame was inserted after a universal CAG promoter.[1] A transcriptional/translational stop region was included to prevent GRP78 expression, which was flanked by two loxP sites. Transgene positive founders were crossed to αMHC-Cre animals to trigger cardiomyocyte-specific expression of GRP78.

Cell surface protein biotinylation. After GRP78 knockdown, NRVMs were washed with cold PBS. EZ-link-Sulfo-NHS-LC-Biotin (Thermo) was added (0.5 mg/mL) for 30 min at 4°C with gently shaking. Tris solution (pH 7.5, 100 nM) was used to quench the reaction. The cells were then washed twice with ice cold PBS and lysed with RIPA buffer. Neutroavidin agarose beads were then mixed overnight at 4°C. After 6 washes, the beads were boiled in SDS-PAGE loading buffer and the supernatants were used for immunoblotting.

Immunofluorescence staining. GRP78 staining was conducted according to a previously reported method with modifications.[2] Briefly, NRVMs were first washed with cold PBS and fixed in 4% paraformaldehyde for 10 min. After blocking with 5% BSA for 30 min, GRP78 antibodies (1:1000) were added to incubate with the cells overnight at 4°C. After washing, the cells were stained with Alexa Fluor-594 goat anti-mouse antibodies without cell permeabilization. Saponin (0.5%) was then used to permeabilize the cell at RT for 1 hr. Primary antibodies for p85 (4292, 1:50, Cell Signaling) were used to incubate with the NRVMs for 2 hrs at RT. After 3 washes, Alexa Fluor-649 goat anti-rabbit antibodies were used to detect p85. The cells were then counterstained with DAPI before confocal imaging. Co-staining for PIP3 was similarly conducted with mouse anti-PIP3 antibodies (Z-P345b, 1:50, Echelon Biosciences Incorporated).

Supplementary references

1. Niwa H, Yamamura K, Miyazaki J. Efficient selection for high-expression transfectants with a novel eukaryotic vector. *Gene* 1991, **108**(2)**:** 193-199.

2. Zhang Y, Tseng CC, Tsai YL, Fu X, Schiff R, Lee AS. Cancer cells resistant to therapy promote cell surface relocalization of GRP78 which complexes with PI3K and enhances PI(3,4,5)P3 production. *PLoS One* 2013, **8**(11)**:** e80071.
